# Accurate whole-night sleep monitoring with dry-contact ear-EEG

**DOI:** 10.1038/s41598-019-53115-3

**Published:** 2019-11-14

**Authors:** Kaare B. Mikkelsen, Yousef R. Tabar, Simon L. Kappel, Christian B. Christensen, Hans O. Toft, Martin C. Hemmsen, Mike L. Rank, Marit Otto, Preben Kidmose

**Affiliations:** 1 Department of Engineering, Aarhus University, Aarhus, Denmark; 2 Department of Electronic & Telecommunication Engineering, University of Moratuwa, Katubedda, Sri Lanka; 3 UNEEG medical A/S, Lynge, Denmark; 4 Department of Clinical Neurophysiology, Aarhus University Hospital, Aarhus, Denmark

**Keywords:** Sleep, Biomedical engineering

## Abstract

Sleep is a key phenomenon to both understanding, diagnosing and treatment of many illnesses, as well as for studying health and well being in general. Today, the only widely accepted method for clinically monitoring sleep is the polysomnography (PSG), which is, however, both expensive to perform and influences the sleep. This has led to investigations into light weight electroencephalography (EEG) alternatives. However, there has been a substantial performance gap between proposed alternatives and PSG. Here we show results from an extensive study of 80 full night recordings of healthy participants wearing both PSG equipment and ear-EEG. We obtain automatic sleep scoring with an accuracy close to that achieved by manual scoring of scalp EEG (the current gold standard), using only ear-EEG as input, attaining an average Cohen’s kappa of 0.73. In addition, this high performance is present for all 20 subjects. Finally, 19/20 subjects found that the ear-EEG had little to no negative effect on their sleep, and subjects were generally able to apply the equipment without supervision. This finding marks a turning point on the road to clinical long term sleep monitoring: the question should no longer be whether ear-EEG could ever be used for clinical home sleep monitoring, but rather *when* it will be.

## Introduction

Sleep monitoring, meaning the recording and subsequent analysis of physiological signals during sleep, is important in the study and diagnosis of a long list of diseases^1^. However, the gold standard method, the polysomnography (PSG) is both an encumbrance for the patient as well as expensive to perform. Therefore, the need for monitoring must always be balanced against practical constraints, and it is rarely feasible to perform more than one or two full-night recordings at a time.

However, in both treatment and study of several diseases, it is very valuable to monitor quality and extent of patient sleep over extended periods (weeks or longer). Important examples are idiopathic hypersomnia and circadian rhythm sleep-wake disorders, but studies such as Ciano *et al*. 2017 and Ye *et al*. 2018 also demonstrate the value of longitudinal sleep monitoring in more general care as well as research^2,3^.

These circumstances (the limitations of the PSG and the need for long term sleep monitoring) fuel an ongoing effort to develop a cheap, precise alternative to the PSG, suitable for long-term monitoring. Of special interest in this endeavor is the method of ear-EEG^4^, in which recording electrodes are placed in or around the ears. This placement is useful because the area is largely hairless and relatively hidden (meaning the possibility of good signals and a low-profile device). Previously, a good correspondence has been shown between ear-EEG and scalp EEG^5,6^.

For this reason, Mikkelsen *et al*. 2017 probed the use of ear-centered EEG in full night recordings, with promising results but a limited number of nights (only 9)^7^. Similar work was done by Nguyen *et al*. and Alqurashi *et al*., but with only nap studies instead of full nights^8,9^. More recently, Mikkelsen *et al*. 2018 and Nakamura *et al*. 2019 performed 15 and 22 full-night recordings using ear-EEG and full PSG, finding increases in performance, but still a significant gap between device and PSG performances^10,11^. All these studies used wet electrodes, which can be difficult for subjects to apply themselves. The performances in all cases were promising but not ideal. Most recently, Mikkelsen *et al*. 2019 showed that the same setup used in this paper can detect sleep spindles (but no sleep scoring was performed)^12^.

**Figure 1:**
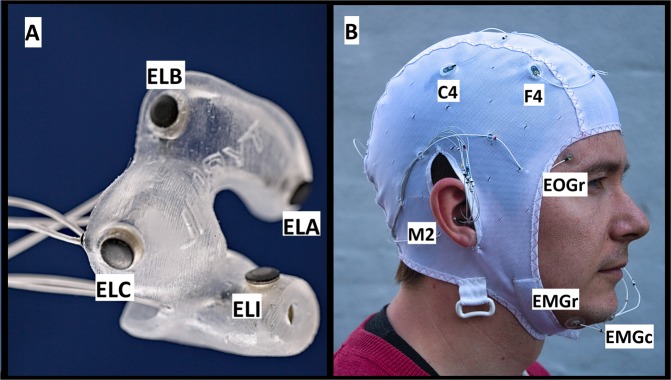
Recording setup: (**A**) Shows a left-earplug with 6 dry electrodes (positions A, B, C, I are visible and labelled). (**B**) Shows the full setup, with both earplugs and EEG cap. Visible PSG electrodes are labelled.

In this study we increased the number of recordings considerably, to 80 recordings from 20 subjects (4 from each), and used dry electrodes, which is a significantly more realistic technology for home use^13^. Each night, subjects wore both ear-EEG and a partial PSG setup (EEG, EOG and EMG electrodes, please see Fig. 1 and the ‘Methods’ section). As in most previous studies, we used a machine learning approach to automatically sleep score the recordings, as that is the most realistic approach for the large amounts of data that would result from a a good home monitoring solution. Importantly, automatic scoring has been found to outperform manual scoring for this data type^10^. What is also new is that we perform multiple repeated recordings on the same subject, to investigate the relative strengths of both broad and narrow training sets.

Users reported acceptable comfort levels, though with room for improvement. After instructions, most subjects could place the ear pieces correctly in their ears without assistance.

We find an over all high quality of recordings, loosing only 4 percent of all epochs.

Most importantly, we show a performance of the automatic classifier at a level very close to the gold standard of manually scored PSGs^14^.

## Results

All 20 subjects finished 4 recordings each, resulting in 657 hours of analyzed recordings, or 78799 epochs (72942 after epoch rejection).

### Comfort and ease of use

**Figure 2:**
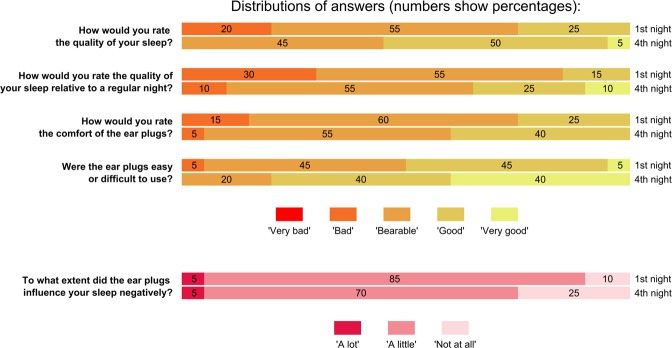
Answers to the sleep questionnaire after each subject’s first and last nights. Note that there are 20 answers given to each question (one for each participant), meaning thath ‘5%’ corresponds to a single participant. For each question, we see a trend of answers moving towards more positive responses.

Figure 2 shows participant answers about device comfort and useability after their first and fourth nights. Overall, when considering the comfort of their sleep measurement, the majority describe the experience as bearable, but with room for improvement. The one exception is ease of use, which seems to already be satisfactory with 80% describing the ear plugs as easy or very easy to use. Across all questions, we see a clear trend of users becoming more positive towards using the equipment as they gain experience with it.

Participants were informed that they could remove the ear pieces in two different situations:

1. The participant could remove the ear pieces temporarily during the time between setup and recording start (putting them back before starting), if they felt it would increase the chances of a comfortable recording. This option was used by 6 subjects a total of 14 times.
2. The participant could remove one or more ear pieces after recording start if they found it necessary to falling asleep. Due to this, on 3 occasions the participant removed a single ear piece during the recording (by 3 different subjects). No subjects found it necessary to remove both ear pieces.

Of the 14 occasions on which a subject put one or more ear pieces back into the ears at home, only at one instance did they fail to obtain the same connection quality as had previously been achieved during set-up. This means that while it was not a primary objective of this study, and the ear plugs have not been optimized for easy mounting, participants were quite good at mounting the ear-EEG without supervision.

As part of the study protocol, subjects were asked regarding ‘adverse events’ after each recording. Except for occasional soreness, the only related event was an incident of skin irritation in one ear canal, which disappeared within 4 days.

### Data quality

**Figure 3:**
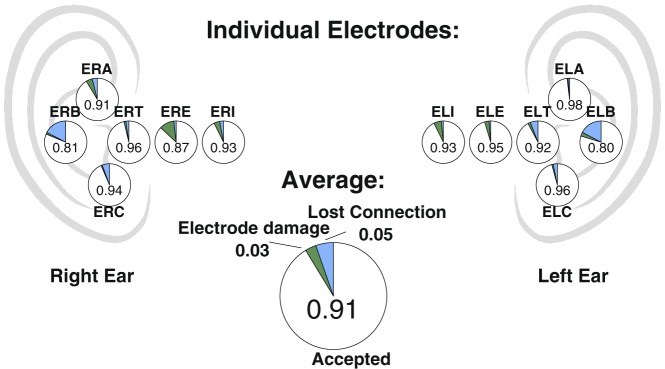
Data quality of individual electrodes: Proportion of accepted data for each of the 12 ear electrodes (written), together with the reasons for data rejection (colored) and an overall average. Each pie chart is placed roughly in the position of the corresponding electrode (name above/below), except that distance into the ear canal is exaggerated.

In Fig. 3 is shown pie charts for each electrode, summarizing performance over all 80 recordings. We see a generally high electrode performance, spanning from 0.80 up to 0.98 sample-wise inclusion fraction, with an average of 0.91. It is interesting to note that the cause of failures varies with location, such that connection loss is the leading cause of failure in the concha region (electrodes A,B,C), while inside the ear canal, the main reason is electrode failure (principally moisture interfering with electrode shielding).

Note that very little of this data rejection has resulted in unscored epochs. Epoch rejection only happened if an entire ear was missing for a full epoch, which mainly occurred when the subject removed the ear piece themselves. 4.3% of epochs have been rejected because an entire ear is missing. Of these, 61% (2.6% of all epochs) were due to earpiece removal by the subject.

Finally, in order to assess the correspondence between scalp-EEG and ear-EEG we used the method described in Mikkelsen, Kidmose and Hansen, 2017^6^ to estimate the mutual information between ear-EEG and the mastoid-to-mastoid derivation. The mutual information, estimated from data recorded during the lab setup, was on average 0.76. Thus, the information carried by the ear-EEG was very similar to the information in the mastoid-to-mastoid derivation.

### Sleep scoring

**Figure 4:**
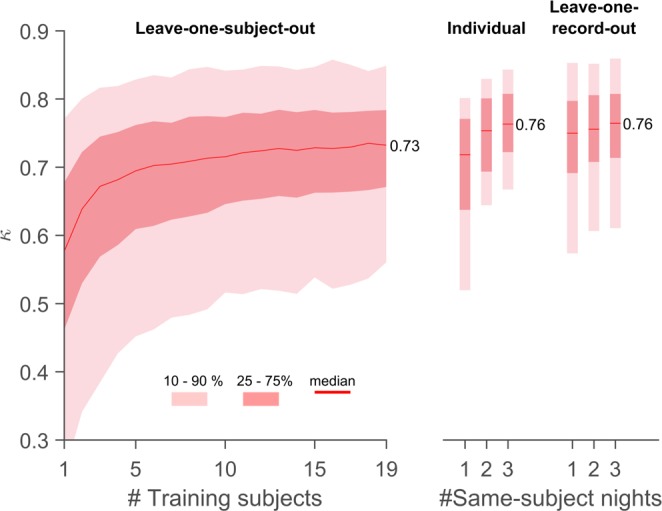
Classifier performance as a function of training data: On the left is shown the distribution of Cohens kappa values for leave-one-subject-out (LOSO) classification versus number of subjects in the training data (N). On the right is shown distributions for ‘Individual’ (using only recordings from the same subject) and ‘Leave-one-record-out’ (LORO (combining recordings from all other subjects with 1, 2 or 3 from the same subject). For LOSO, each value of N was repeated 100 times, to obtain unique samplings of training subjects. All subjects not used for training were used for testing, meaning that the number of kappa values for a given N was (20-N) times the number of repetitions. For N = 1 and 19, only 20 repetitions were done (being the number of unique combinations). For Individual and LORO, all possible combinations were used (resp. 80, 120, 80 combinations for 1, 2 or 3 nights).

Using 30-second epochs, the sleep recordings were scored both manually and using a machine learning approach (manual scoring based only on PSG electrodes, automatic scoring only on ear-EEG electrodes). Figure 4 shows distributions of Cohen’s kappa between these two sources of scoring, for different ways of training the automatic scoring algorithm. The training methods differ by how test- and training data was chosen (see the methods section for a detailed description). Starting on the left, we see that the performance increase in leave-one-subject-out (LOSO) performance when going from 10 to 19 training subjects (corresponding to 40 and 76 nights) is limited, and also that the ‘bottom’ of the distribution requires more data to converge than the top (which is largely unchanged at 5 subjects and above). Moving on to the invididualized models, reported in the middle, we see an impressively high performance after 3 nights’ training, with clear performance increases between both 1 and 2 and 2 and 3 nights. It would be interesting to see how long that trend would have continued with more nights. Comparing the ‘individual’ results with the LOSO results, we see that the LOSO models do not reach the full potential of the ear-EEG method, missing out on some subject-specific sleep information in the recordings. Finally, leave-one-record-out (LORO) on the right shows us that the benefit of subject-specific data is compatible with the broad classifier from LOSO. We interpret this to mean that the performance increase when going from LOSO to ‘Individual’ is not due to a decrease in ‘noise’ but rather a more relevant coverage of the feature space. This would, in turn, mean that the LOSO performance is not yet converged at 19 subjects, but could conceivably reach similar performance to what is seen for LORO with sufficient training data.

**Figure 5:**
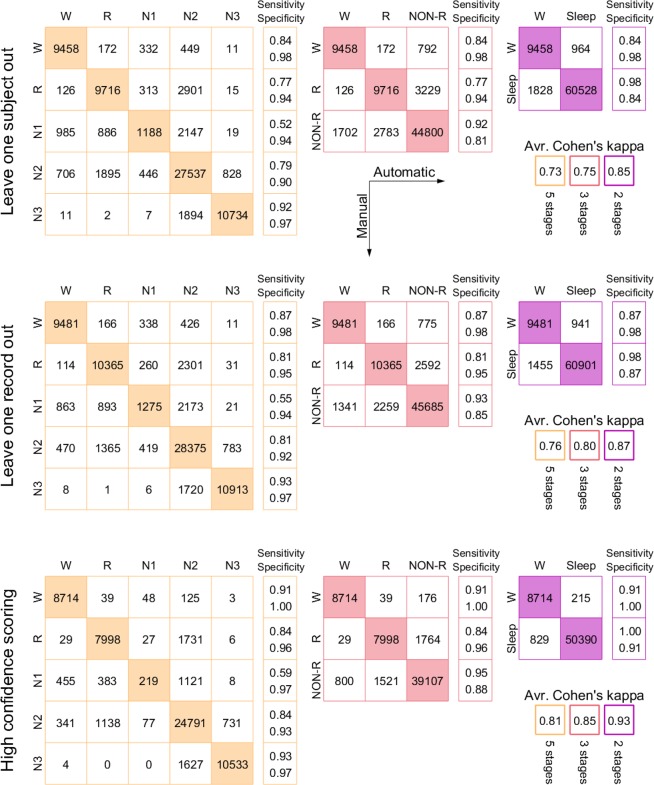
Confusion Matrices: Comparison between manual scoring and the results from 3 different methods of classifier training, for different ways of scoring the recordings. Each describes results for 5, 3 and 2 sleep stages (in the second, N1, N2 and N3 are grouped, in the last, REM is added to that as well). Numbers signify epoch counts. In the top row, the classifier was trained on all recordings from all other study participants. In the middle row, it was trained on all other recordings in total (including the other three from the same participant). In the bottom row, the classifier was trained on all recordings from all other subjects (like the top row), but in the output only those epochs which had greater than 50% confidence were included. This amounted to excluding 18% of epochs. On the right is shown average Cohens kappa (averaged over all recordings). Note that the 3 and 2-stage scorings were computed by grouping the 5-stage scoring, not by retraining classifiers.

Figure 5 shows confusion matrices for 3 different methods of cross validation, LOSO, LORO and the special case where only epochs with confidence greater than 0.5 are included (excluding 18% of epochs). This third case is based on leave-one-subject-out. Except for the usual case of N1 which is notoriously hard for scorers to agree on^14,15^, we see generally very high levels of sensitivity and specificity. In addition, we see that practically all errors are either misclassifications of N1 or involving N2 - there is nearly no confusion between R, N3 and wake.

In Fig. 5 is also shown average kappa across recordings (Fig. 4 only shows median), in particular the 5-stage LOSO value is 0.73. For comparison, LOSO performance as reported in^7^ and^10^ was 0.44 and 0.56, while Cohens kappa for two scorers from different sleep centers was found in^14^ to be 0.76. In other words, the performance demonstrated here is considerably better than what has been seen before, and approaching the practical limit given by inter-scorer agreement.

**Figure 6:**
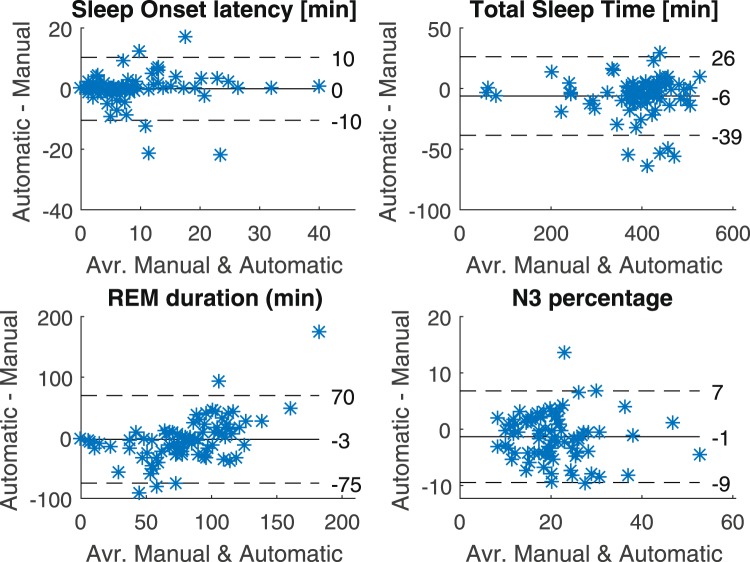
Sleep metrics: Bland-Altman plots for four different sleep metrics, comparing manual PSG scoring and automatic earEEG scoring (leave-one-subject-out). In each plot is included the mean (full line) as well as the mean ±1.96 standard deviations. Only epochs that could be scored by the sleep technician, as well as by the algorithm (meaning it had functional electrodes in both ears) were included - the rest were marked as ‘unscored’ in both hypnograms.

Finally, in line with the generally high kappa values, we find that the automatic scoring does well in reproducing key sleep metrics, calculated from the hypnograms. Figure 6 shows Bland-Altman plots for 4 different sleep metrics, and we see a generally high level of correspondence between the manually and automatically generated hypnograms.

## Discussion

### Comfort

In total, 80 + 1 full night recordings were performed. The extra night was performed because in one case, technical issues not related to the ear-EEG were so severe as to make the manual scoring impossible, and that recording was redone. To avoid any bias caused by this, the comfort analysis for this participant was still based on the person’s first four nights (as if nothing had been redone).

When considering the comfort results, it is important to note that we did not ask the subjects to compare the ear-EEG and PSG equipment, nor did we probe the comfort of the PSG setup itself. There were multiple reasons for this decision, the primary one being that the ‘partial PSG’ setup used here deviates somewhat from the standard setup with disposable electrodes used in many sleep clinics, reducing the relevance of the comparison.

When considering the comfort of ear-EEG (or any new sleep monitoring device), one thing is important to consider: the standard setup is by no means unobtrusive itself. This in turn means that a solution for long-term monitoring does not itself have to be completely unfelt to deliver meaningful sleep data. Finally, we should mention that several subjects, on their own volition, either on the sleep questionnaire or verbally, expressed the view that the ear-EEG was *less* intrusive than the rest of the setup. Future studies will examine the perceived comfort of PSG-free nights, and compare them to the findings presented here.

During the study, it was discovered that the depth of each ear plug (i.e. how far into the ear canal the silicone extended) was closely related to wearer comfort, with only minor influence on data quality. In one subject, #2, it was decided after a very poor first night to reduce the ear-piece depth through a redesign. This resulted in a marked increase in comfort, but was only done for that subject. Note that the subject indexing is based on an alphabetical ordering of subject names (not chronology).

### Sleep scoring

As discussed above, the performances of both LORO, ‘Individual’ and LOSO cross validations are considerably better than what has been shown previously for ear-EEG, and are approaching the inter-scorer values, which could reasonably be considered the practical max^14^.

We can think of one important reason why the real gap between the inter-scorer kappa values and those presented here may be slightly larger in reality. The single-scorer setup used here allows the classifier to learn and capitalize from any ‘bad habits’ on the part of the scorer. Such habits would otherwise work to decrease the kappa value in an inter-scorer comparison. This means that if a study like^14^ was to be conducted today, with an ear-EEG based automatic classifier as one of the scorers, the performance gap between the ear-EEG based scoring and the rest would likely be slightly larger than the gap we find here. Of course, these considerations are also applicable for^7^ and^10^, meaning that the size of the performance jump is unaffected.

**Figure 7:**
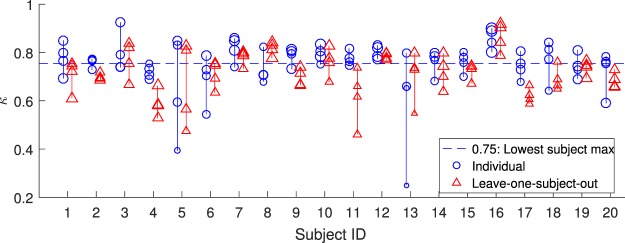
Subjectwise performance: Each marker corresponds to a single night, with the kappa-value calculated between manual scoring and the output of either an individualized or leave-one-subject-out automatic model. Lines connect circles from the same subject (same subject ID). While a few subjects present some nights which are difficult to score, all subjects present at least one night which is very easy to score for the individual model (with a kappa value at 0.75 or greater). Marker size reflects median label confidence.

As evidenced in Fig. 7, all subjects have at least one night for which the individual classifier attains a Cohen’s kappa value equal to or greater than 0.75. We interpret this to mean that there are no subjects for which the ear-EEG in itself is a bad approach, instead some subjects had one or more unfortunate recordings. This interpretation is strengthened when we consider the details of the recordings themselves. Starting from the bottom, we find that the two hardest-to-score nights, with ‘Individual’ kappa values of 0.25 and 0.39, both suffered from distinct practical issues: in one case 5 ear-electrodes had moisture-damage, in another the subject removed the earplug prior to recording start and subsequently failed to put it back correctly.

We observe in Fig. 4 that while there is a performance increase when moving from ‘leave-one-subject-out’ (LOSO) to ‘Individual’ classifier training, this gain is preserved when the individual and general data sets are combined in ‘leave-one-record-out’ (LORO). We interpret this to mean that the issue which is solved when going from LOSO to ‘Individual’ is a too sparse coverage of feature space, rather than different subjects confounding the classifier. In other words, when the classifier does not perform optimally, it is because it has not been trained on sufficiently similar data to the testing data; it is *not* because the same ear-EEG data may mean different things for different subjects. This, in turn, means that it should be possible for LOSO to attain the same high performance as LORO, if the testing population is sufficiently well covered by the training data.

**Figure 8:**
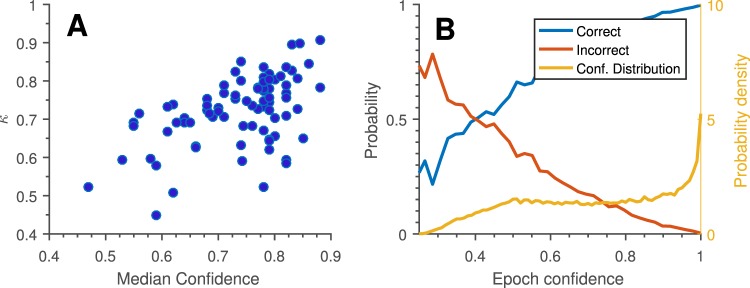
Classifier performance versus classifier confidence: The automatic sleep scoring algorithm also estimates the probability of the chosen label being correct. This may be interpreted as our ‘confidence’ in the scoring. (**A**) Median confidence for all assigned labels across each night relative to Cohen’s kappa for the same night. We see a clear trend of greater confidence across a whole recording corresponding to greater overall scoring performance. (**B**) Observed probability of a given epoch being correctly/incorrectly labeled, as a function of label confidence (plotted to left axis). ‘Conf. Distribution’ shows the distribution of epoch-level confidence estimates (plotted to right axis). As in (**A**), we see a clear trend of high confidence epochs generally being correctly labelled. Given an epoch of specific confidence we see that confidence has to be below 0.4 for the label to be most likely incorrect, and that most confidence estimates are above 0.5. Both graphs are based on leave-one-subject-out cross validation for 5-stage scoring.

It is very interesting and useful to see the good correspondence between classifier confidence (the estimated probability of the label being correct) and classifier performance, evidenced in Fig. 8. This correspondence means that it is possible without any outside information to estimate the likely quality of the scoring. In turn, this would enable a clinician to decide whether the data obtained from the ear-EEG for a given patient was of sufficient quality, or whether additional nights were needed. This is effectively the best of both worlds: possibility of low-cost, low intrusion monitoring combined with easy control of data quality.

We have not been able to show a dependence of ‘Individual’ model performance on total participation duration; i.e. we do not see that subjects with large delays between recordings are harder to score than subjects with all recordings done in a short time frame. We find it likely that such an effect would become visible with more subjects (for now, the inter-night variation is dominated by recording issues such as moisture).

Finally, comparing ear-EEG performance to that of commercially available sleep scoring devices, we are aware of two candidates with credible clinical studies, the ‘Sleep Profiler’^16^ and the ‘Dreem’ headband^17^. We find that in terms of average Cohen’s kappa, the ear-EEG compares favourably to the former, and seems mostly indistinguishable from the latter. We suspect the optimal choice of device in that case would depend on comfort, ease of use and any future developments of automatic algorithm for either device.

## Conclusion

We find that automatic scoring of sleep using ear-EEG can perform at levels almost identical to expert sleep stage scoring of PSG, in young healthy subjects.

On this basis, we propose that the question should no longer be *whether* a mature ear-EEG sleep monitor could one day be used for long term medical sleep monitoring, but rather *when*, and what the benefits will be.

Given the present performance and general design, we anticipate that ear-EEG can make a valuable contribution in the treatment and diagnosis of sleep disorders in which it is the amount, timing or type of sleep which is of interest, such as narcolepsy, insomnia or idiopathic hypersomnia.

We see every reason to continue investigating the possibilities and limitations of ear-EEG sleep monitoring, both in terms of increasing comfort, using generic ear pieces, and deployment in patient populations. This includes a separate analysis of single-ear sleep scoring, since it was found that all subjects could sleep well when wearing only one ear piece.

Of particular interest is the clinical value of easy access to several nights of recordings for all patients, rather than single nights, and to which extent the ear-EEG will be more or less susceptible to the signal degradation associated with important sleep-affecting illnesses, such as dementia, insomnia, depression or post-traumatic-stress-disorder (PTSD).

## Methods

### Recordings

20 subjects were recruited to the study, of which 13 were female. The ages of the subjects ranged from 22 to 36, with a mean of 25.9 years. Participants were screened for hearing loss, sleep disorders, neurological disorders, bruxism, pregnancy, drug usage, allergies, chronic pain and sleep apnea. In relation to sleep apnea, all subjects also answered the STOP-BANG questionnaire^18^, scoring a 1 or less.

The study was approved by the Central Denmark Region Committees on Biomedical Research Ethics (Ref. nr. 1-10-72-413-17) as well as the Danish Medicines Agency (ref. nr. 2017111085). Additionally, the study was registered with clinicaltrials.gov, with the protocol ID ‘EarEEGSleep2018’. The study was monitored by the ‘Good Clinical Practice’ unit at Aarhus University, and all methods were performed in accordance with the relevant guidelines and regulations. All participants provided written, informed consent before any action was taken, and exclusion criteria were re-checked at every visit.

Informed consent was obtained from any individual for the publication of their image.

Custom-fitted earpieces were made for all subjects from personal ear impressions. Subsequently, 4 nightly recordings were performed for each subject, each including both the partial PSG and ear-EEG. After each recording, the subject was tasked with filling out a sleep diary, including times of recording start, lights out, awake times during the night and a questionnaire on comfort. Subjects also wore an actigraphy device with event logging, which made accurate event times possible.

The time between measurements on the same subject was on average 19 days, with a large standard deviation of 18 days (25% of recording intervals were at or below 1 week, 51% were at or below two weeks).

Setup was performed in an EEG laboratory except for a single subject whose setup was done at home. Electrode connections were tested using visual inspection of jaw clench responses and measurement of an auditory steady-state response. All recordings took place at the subjects’ own home.

Subjects were informed that if they felt particularly bothered by the earpieces, they were allowed to remove them during the afternoon and evening and reinsert them before recording start, but needed to report it in their sleep diary.

Additionally, the subjects were given the option to remove one or both ear pieces during the recording if the discomfort from wearing them became too great, or if they were unable to fall asleep with both ear pieces in the ears (which could keep some subjects from lying on their side).

#### Recording setup

All electrodes were iridium oxide, as described in Kappel *et al*.^13^. The partial PSG setup was a combination of face electrodes in soft silicone holders and an EASYCAP EEG cap (EASYCAP GmbH, Germany), modifed in-house. See Fig. 1b for a visualization. The PSG setup consisted of two EOG electrodes, 3 chin EMG electrodes and 8 EEG electrodes: M1, M2, F3, F4, C3, C4, O1, O2 electrodes (according the 10–20 system). This was all done to comply with the ASSM standard, and allow manual sleep scoring of the recordings by a trained professional. M1 and M2 electrodes were placed directly on the skin with a bit of Ten20 conductive electrode gel (Weaver and Company, USA), and F3, F4, C3, C4, O1, O2 electrodes had Elefix (Nihon Kohden, India) gel applied to ensure stable connection through the hair.

Ear-EEG ear pieces were custom designed for each participant, and molded in Detax Softwear 2.0 shore A 60 “earmould silicone” (Detax, Germany). Each ear piece contained six electrodes: A, B, C, T, E, I, according to the nomenclature set out in^4^, with the change that the ‘T’ electrode was placed on the tragus, and that the ‘E’ and ‘I’ electrodes were named according to their distance to the ‘T’-electrode, with ‘E’ being closest. See Figs 1a and 3 for a visualization.

Both PSG and ear-EEG electrodes were connected to a Mobita mobile amplifier (TMSi, The Netherlands), with a common ground electrode placed on the neck (disposable Ag/AgCl ECG electrode). The EEG was sampled at 500 Hz.

During the recording, participants wore a GENEActiv actiwatch (Activinsights, UK), sampling at 100 Hz, which allowed accurate reporting of recording events.

### Detailed recording procedure

#### Preparation and quality check

At the start of each recording setup, the subject was questioned regarding the screening criteria, and any adverse effects since the last visit.

Prior to attachment of M1, M2, EOG and EMG electrodes, the skin was cleaned with an alcohol wipe. After attachment of mastoid electrodes, the participant was asked to insert ear plugs themselves, with the investigator controlling the final positioning.

After all electrodes had been placed and gel applied, jaw clench responses were monitored live. When it was clear that all electrodes had jaw clench responses, an auditory steady-state response (ASSR) paradigm was performed:

ASSR recording was performed using a broadband chirp stimulus^19^ presented with a repetition rate of 40 Hz. The sound stimulus was presented binaurally at 40 dB relative to sensation level for 4 minutes. The ASSR SNR was determined in the frequency domain as the ratio between the amplitude at the repetition rate (S) and the average noise (N) amplitude ±10 Hz relative to the repetition rate^20^.

Based on the ASSR recording, the signal-to-noise-ratios of the ASSR response were calculated, and the automatic artefact rejection algorithm (described below) was run. Based on these results, it was decided whether any electrodes needed further attention.

At the end of setup, participants were instructed in the use of the amplifier, actigraphy device, sleep diary and given tips on how to sleep comfortably with the equipment.

The time from lab setup to recording start was on average 5 hrs and 9 minutes, with a standard deviation of 2 hrs and 27 minutes.

#### Home measurement

Approx. 30 minutes before going to bed, the participant would start the actiwatch and the Mobita, and create a synchronization point for the two data sets (by shaking the devices in unison). During the night, extended awake times and other events of interest were marked in the sleep diary (with accompanying marks on the actiwatch).

After each measurement, all electrodes were rigorously tested for wear and tear, and were either repaired or replaced if any change in performance was detected.

### Manual scoring

All recordings were scored by the same professional sleep scorer, according to version 2.5 of the American Academy of Sleep Medicine Manual for the Scoring of Sleep and Associated Events^21^. The scorer did not have access to any data from the ear-EEG.

In 3.8% of epochs, the scorer was not able to make a confident decision. These epochs were excluded both from the training and test sets.

### Data preprocessing

After recordings had been extracted from the amplifier, DC-values were deducted from each channel, the channels were bandpass-filtered to 0.1–100 Hz, resampled to 250 Hz, and artefacts were identified and removed from the recording.

#### Artefact rejection

The ear-EEG recordings were subjected to an artefact rejection pipeline with multiple steps:

**Out-of range**: If an electrode left the dynamic range of the amplifier, the reading was replaced with a ‘NaN’-value, and hence automatically discarded upon loading.

**Compromised shields**: The Mobita amplifier employs active shielding of each electrode, in order to reduce external interference and eliminate capacitive loading of the electrophysiological signal. However, due to moisture or other contaminants, the insulation between the inner core and the shield might be compromised, and the recorded signal from the affected electrodes can be altered. This was determined to be the case if multiple EEG channels were exactly equal or if one or more channels had no high-frequency content. Any electrode exhibiting this behaviour was removed entirely from the recording.

**Notch filtering**: All channels were notch filtered at 50 and 100 Hz.

**Spike rejection**: Occasionally a single electrode may move slightly relative to the skin, resulting in a brief, large amplitude deviation in a single channel.

**Electrode rejection**: The signal from a single electrode may, either due to a mechanical defect or suboptimal skin connection, be dominated by high frequency (>20 Hz) noise related to the instability of the connection. Such episodes were defined to have a duration of at least 30 seconds, and were identified by thresholding the envelope of the high-frequency portion of the signal.

**Movement and EMG rejection**: Subject movement or muscle activation result in large amplitude deviations across multiple channels. If at least 20% of active ear-EEG channels exhibited such behaviour simultaneously, all channels were rejected in that period (using epochs of 1 second duration). Unlike the other rejection criteria, this information was passed on to the automatic sleep scoring algorithm, in the form of how much movement was rejected in a given sleep scoring epoch.

**Miscellaneous**: At the end, any samples with absolute value greater than 350 *μ*V were discarded. This happened for less than 0.1% of samples.

In Fig. 3, for clarity, we have pooled ‘Out-Of-Range’, ‘Spike rejection’ and ‘Miscellaneous’ under the term ‘Lost Connection’, while ‘Compromised shield’ and ‘Electrode rejection’ have been pooled under ‘Electrode damage’. ‘Movement and EMG’ were excluded, since we consider this to be an issue with the EEG method in general and not related to the ear-EEG method. In addition, since the movement information is passed on to the classifier, such epochs are usually still possible to sleep score correctly for the automatic classifier. On average, 6% of a recording is flagged as movement and muscle activation.

Rejected samples were replaced by ‘NaN’-values.

#### Epoching

After data rejection, each recording was partitioned into 30-second non-overlapping epochs. If an entire ear was missing (due to other causes than extended Movement/EMG artifacts), for an entire epoch, that epoch was excluded from further analysis.

#### Feature extraction

We computed the 33 features described in^7^, originally inspired by^22^. We also included 44 new features based on Continuous Wavelet Transform (CWT) an 6 new non-linear features. For completeness, we have included a list of all features, see Table 1, and a detailed description of all non-trivial features in the Supplementary Methods.

**Table 1:** An overview of the features used in this study, grouped by type. See the Supplementary Methods for a detailed description of all non-trivial features.

Label	Short Description	Type
F1	Signal Skewness	EEG Time Domain
F2	Signal Kurtosis	
F3	Zero Crossing Rate	
F4	Hjorth Mobility	
F5	Hjorth Complexity	
F6	75th percentile	
F7	Channel Correlation	
F8	EMG power	EMG proxy
F9	Minimal EMG Power	
F10	Relative EMG burst amplitude	
F11	Slow Eye Movement power	EOG proxy
F12	Rapid Eye Movement power	
F13- F16	Relative power in *α*, *β*, *θ*, *δ*-bands	EEG Frequency Domain
F17-F23	Power-ratios: *δ*/*θ*, *θ*/*α*, *α*/*β*, *β*/*γ*, (*θ* + *δ*)/(*α* + *β*)	
F24	Spectral edge frequency	
F25	Median power frequency	
F26	Mean spectral edge frequency difference	
F27	Peak power Frequency	
F28	Spectral Entropy	
F29	Spindle probability	Sleep event proxies
F30	Frequency stationarity	
F31	Lowest adj. frequency similarity	
F32	Maximum B-spline transform	
F33	Longest sleep spindle	
F34-F38	Power mean in *γ*, *α*, *β*, *θ*, *δ* bands	CWT based features
F39-F43	Power variance in *γ*, *α*, *β*, *θ*, *δ* bands	
F44-F48	Power skewness in *γ*, *α*, *β*, *θ*, *δ* bands	
F49-F53	Power kurtosis in *γ*, *α*, *β*, *θ*, *δ* bands	
F54-F58	Power entropy in *γ*, *α*, *β*, *θ*, *δ* bands	
F59-F63	Duration of the activation in *γ*, *α*, *β*, *θ*, *δ* bands	
F64-F68	75 percentile in *γ*, *α*, *β*, *θ*, *δ* bands	
F69-F73	Relative power in *γ*, *α*, *β*, *θ*, *δ* bands	
F74-F77	Power ratios: *δ*/*θ*, *θ*/*α*, *α*/*β*, *β*/*γ*	
F78-F82	Multi scale entropy 1–5	Non-linear features
F83	Lempel-Ziv complexity	

All features were calculated based on 3 different electrode derivations: 〈left ear〉 − 〈right ear〉 and 〈concha〉−〈canal〉 for each ear, where the concha electrodes are ‘A’, ‘B’, ‘C’ and canal are ‘T’, ‘E’, ‘I’ and 〈·〉 signifies the average, ignoring ‘NaN’-values. If the resulting derivations had NaN-values (for instance during movement artefacts where all electrodes are excluded simultaneously), the samples were interpolated using the closest non-NaN values on either side (time wise). For long interpolation segments, the synthetic data was made to converge to zero far from the edges. Please note that this does not introduce any new information to the classifier.

### Classifier training

Each 30-second epoch was described by the 83 features as listed in Table 1, calculated for the three derivations described, for both the epoch itself, the 4 epochs preceding it and the 2 following it (7 epochs in total). In addition, the classifier was also informed how much of each epoch was removed due to EMG and movement artefacts. In total, this means each epoch was described by 1750 numbers: 1750 = 7 epochs · (3 derivations · 83 features + 1 estimated movement time).

When evaluating the automatic scoring, test and training sets were chosen in three different ways:

**Leave-one-subject-out (LOSO)**: When scoring a night from subject *i*, the classifier was trained using all four recordings from N (between 1 and 19) other subjects. Thus, the classifier has not been trained on data from the subject on which it is tested.

**Individual (IND)**: When scoring a night from subject *i*, the classifier was trained using only 1, 2 or 3 recordings from subject *i* (but not the night being scored). Thus, no data from other subjects were used during training.

**Leave-one-record-out (LORO)**: When scoring a night from subject *i*, the classifier was trained using all recordings from other subjects, combined with 1, 2 or 3 recordings from subject *i*.

The classifier consisted of an ensemble of 100 decision trees, forming a so-called random forest^23^. This was chosen because both the authors and other groups have previously found random forests in particular to be well suited for classification of EEG data^7,10,24^, and it works well with the amount of data available (individual models would be difficult to implement with neural networks, for instance). An important strength of random forests is that they are resistant to overfitting, even in a high dimensional feature space. This is achieved by creating an ensemble of different classifiers. For an in-depth discussion of this, see^25^.

The decision trees were generated such that each split maximized the Gini coefficient, and continued until each subgroup was homogeneous. The data sets for each tree were chosen using ‘bagging’^26^.

### Cohens kappa

Classifier performance was quantified using Cohens kappa^27^, calculated between the automatic and manual scoring. Cohens kappa rescales observed correspondence with that expected from chance, outputting values upper bounded by 1:

1 $$\kappa =\frac{{a}_{obs}-{a}_{{\exp }}}{1-{a}_{{\exp }}},$$

where *a*_*obs*_ and *a*_*exp*_ are the agreement rates observed and expected from chance.

The Cohens kappa has no theoretical lower limit, but in reality negative values are very unlikely, as are values above 0.9.

For each scoring, we calculated the kappa value in the following way:

Let **M** be the confusion-matrix between manual and automatic scoring, and *n* the number of epochs for the given recording. Letting **s**_*r*_,**s**_*c*_ be the row and column sums of **M** (with one entry for each possible label), then the *i*’th entries of **s**_*r*_/*n*,**s**_*c*_/*n* are the probabilities of a random epoch being given label *i* by each scoring. In turn, the expected number of agreements for label *i* is

$${{\bf{e}}}_{chance}(i)=n{{\bf{s}}}_{r}(i){{\bf{s}}}_{c}(i)/{n}^{2}={{\bf{s}}}_{r}(i){{\bf{s}}}_{c}(i)/n,$$

and the expected number of chance agreements across all labels is

$${e}_{tot}={{\bf{s}}}_{r}(i)\cdot {{\bf{s}}}_{c}(i)/n={a}_{{\exp }}n.$$

*a*_*obs*_*n* = *trace*(*M*) is the sum of all correct labels, so, by substituting *a*_*obs*_, *a*_*exp*_ in (1):

2 $$\begin{array}{rcl}\kappa  & = & \frac{trace(M)/n-{{\bf{s}}}_{r}(i)\cdot {{\bf{s}}}_{c}(i)/{n}^{2}}{1-{{\bf{s}}}_{r}(i)\cdot {{\bf{s}}}_{c}(i)/{n}^{2}}\\  & = & \frac{trace(M)-{{\bf{s}}}_{r}(i)\cdot {{\bf{s}}}_{c}(i)/n}{n-{{\bf{s}}}_{r}(i)\cdot {{\bf{s}}}_{c}(i)/n}\mathrm{.}\end{array}$$

Which is how Cohens kappa is calculated in this study.

## Supplementary information

Supplementary Methods

## Data Availability

Given the very high quality and multitude of possible uses for this data set, the authors intend to make it available for the scientific community (including the software routines described here). However, to comply with the regulations of the governing ethics committee, the data can not be shared without completely anonymizing all copies of the data set, both those publicly available and not. This, in turn, can not be done until the planned follow-up study has been performed, which is expected to happen with one year’s delay relative to this submission. Until then, the authors are happy to share both the aggregate statistics presented in this manuscript, as well as the entire code base.
