# Supplementary material for: Accurate whole-night sleep monitoring with dry-contact ear-EEG
Source: Sci Rep. 2019 Nov 14;9:16824. doi: 10.1038/s41598-019-53115-3 (PMC6856384; doi:10.1038/s41598-019-53115-3)
Supplement: Supplementary file 1 — Supplementary Methods [file 41598_2019_53115_MOESM1_ESM.pdf]

Accurate whole-night sleep monitoring with dry-contact ear-EEG

Kaare B. Mikkelsen[1,*], Yousef R. Tabar[1], Simon L. Kappel[1,2], Christian B. Christensen[1], Hans O. Toft[3], Martin C. Hemmsen[3], Mike L. Rank[3], Marit Otto[4], and Preben Kidmose[1,*]

[1]*Department of Engineering, Aarhus University, Denmark*
[2]*Department of Electronic & Telecommunication Engineering, University of Moratuwa, Sri Lanka*
[3]*UNEEG Medical A/S, Denmark*
[4]*Department of Clinical Medicine, Aarhus University, Denmark*
[*]*Corresponding authors: mikkelsen.kaare@eng.au.dk, pki@eng.au.dk*

October 7, 2019

A Supplementary Methods: Feature description

In the following, the estimated power spectral density will be known as 'P', and $P(f)$ or $P([a,b])$ will be taken to mean the estimated power at frequency f, or the total power in frequency band a to b Hz.

A.1 EEG time domain proxies

F1 - Signal Skewness: Skewness is the 3rd order moment of the signal which measures the asymmetry of the signal's probability distribution about the mean. Signal skewness is expected to be lower in deep sleep.

F2 - Signal kurtosis: Signal kurtosis is a measure of the 'tailedness' of the signal, also known as 4rd order moment of the signal. This feature is also a widely used feature to discriminate between sleep stages.

F3 - Zero crossing rate: Number of times the signal crosses the reference line. Zero crossing rate is known to be quite useful in detection of transient waves like sleep spindles.

F4 - Hjorth mobility: The ratio between the standard deviation of the signal and its first derivative. This feature provides information about frequency content of the signal and the dominating frequency components.

F5 - Hjorth complexity: This feature measures how similar the shape of a signal is to a pure sine wave.

F6 - 75th percentile: The value below which exactly 75% of the measured values fall.

F7 - Channel correlation: This feature measures the Pearson correlation coefficient between ear-EEG derivations. It is the only feature which requires multiple channels and is calculated for all pairs of derivations (in our case there are 3 derivations).

A.2 EMG proxy

These features are calculated based on a 32-80 Hz band-pass filtered version of the original signal.

F8 - EMG power: Total power in the 32-80 Hz band. This is useful in identification of the deeper levels of sleep where there is less high frequency activity.

F9 - Minimal EMG power: This was calculated by dividing each epoch into 10 segments and finding the minimum integrated EMG power among these segments.

F10 - Relative EMG burst amplitude: Is calculated by dividing Maximum EMG signal amplitude by F9.

A.3 EOG proxy

F11 - Slow eye movement power:

$$F11 = \frac{P([0.5, 2])}{P([0.5, 30])}$$

F12 - Rapid eye movement power:

$$F11 = \frac{P([2, 5])}{P([0.5, 30])}$$

A.3.1 EEG Frequency Domain

In the following, the $\alpha, \beta, \theta, \delta$ bands are defined as, 8-16 Hz, 16-32 Hz, 4-8 Hz and .5-4 Hz, in accordance with [1]:

F13-F16 - Relative power in $\alpha, \beta, \theta, \delta$ bands: The power in each band related to the total power in the 2-32 Hz frequency band.

F17-F23 - Power ratios: δ/θ, θ/α, α/β, β/γ, $(\theta + \delta)/(\alpha + \beta)$: The relative power between the specified frequency bands.

F24 - Spectral edge frequency: The frequency below which 95% of the spectral power in the 2-32 Hz band is located.

F25 - Median power frequency: Is the frequency below which 50% of the spectral power in the 2-32 Hz band is located.

F26 - Mean spectral edge frequency difference: The difference between spectral edge frequency (F24) and median power frequency (F25). This feature is successful in separating the REM stage. $F26 = F24 - F25$

F27 - Peak power frequency: The frequency with highest power in the 2-32 Hz band.

F28 - Spectral Entropy: Spectral Entropy measures the disorder in the power spectral density of the 2-32 Hz frequency band. This feature was calculated as:

$$F28 = -\sum_i P(f_i) \ln(P(f_i)) \ ,$$

with f_i running over all frequency bins P.

A.4 Sleep event proxy

F29 - Spindle probability: This feature introduces a spectral mean frequency measure, inspired by [2]. It is calculated as

$$F29 = \frac{\max\left(P\left(11-16\right)\right)}{\left\langle P\left(4-10\right)\right\rangle + \left\langle P\left(20-30\right)\right\rangle}$$

where P(x-y) is the set of power estimates for the [x, y] Hz frequency band.

F30: Frequency stationarity: Each epoch was divided into 31 segments and the periodogram was calculated for each segment. Then, frequency stationarity was computed as the average Pearson correlation between these 31 spectra.

F31 - Lowest adj. frequency similarity: Using the same 31 spectra from F14, F15 was calculated as the lowest Pearson correlation between neighboring segments.

F32 - Maximum B-spline transform: Maximum absolute value of a continuous wavelet transform of the EEG signal. The wavelet type is complex B-spline wavelet with a support of 0.5 s. This feature is successful in detecting sleep spindles and inspired by [3].

F33 - Longest sleep spindle In order to compute this feature, a spindle detection method inspired by [4] was employed. In this method, Teager Energy Operator (TEO) was applied to the 11-16 Hz band pass filtered signal. Concurrently, $P([12, 14])/(P([4, 11])+P([13, 32]))$ was computed by using a Short Time Fourier Transform (STFT) applied to the unfiltered data. Finally, a

wavelet transform (WT) using B-spline wavelet like F32 was performed. The segments of signal in which WT $> 15, TEO > 0.5$ and the STFT power ratio > 0.3 were detected as sleep spindles. F33 was computed as the longest length of the detected spindles.

A.5 CWT based features

All the features in this section were computed using the Continuous Wavelet Transform (CWT) with a Morse wavelet with parameters (3,60). The traditional frequency bands were defined as δ: 0.5-4 Hz, θ: 4-8 Hz, α: 8-12, β: 12-32 Hz and γ: 32-100 Hz.

F54-F58 - Power entropy in $\gamma, \alpha, \beta, \theta, \delta$ bands: The Shannon entropy of the traditional frequency bands.

F59-F63 - Duration of the activation in $\gamma, \alpha, \beta, \theta, \delta$ bands: Duration of the activation feature, defined as the period where the mean power in each frequency band is higher than a threshold. This threshold is selected as $1.5 \cdot$ (median power in 0.5-100 Hz).

F64-F68 - 75 percentile in $\gamma, \alpha, \beta, \theta, \delta$ bands: The frequency below which 75% of the spectral power in each band is located.

F69-F73 - Relative power in $\gamma, \alpha, \beta, \theta, \delta$ bands: The ratio of mean power in each band to mean power in 0.5-100 Hz.

F74-F77 - Power ratios in $\gamma, \alpha, \beta, \theta, \delta$ bands: The ratio of mean power in neighbouring frequency bands.

A.6 Non-linear features

F78-F82 - Multi scale entropy 1-5: Multi Scale Entropy (MSE) is based on sample entropy to compute the entropy over multiple time scales [5]. This feature has been used in several sleep staging studies before [6, 7]. MSE was computed for 5 scales in this study resulting in features F78-F82.

F83 - Lempel-Ziv complexity: Lempel-ziv complexity (LZC) is a nonparametric complexity measure that has been applied to solve many different problems including sleep stage classification [8, 9]. It has been found to be effective in separating N1 and REM stages [8].

References

1. Mikkelsen, K. B., Villadsen, D. B., Otto, M. & Kidmose, P. Automatic sleep staging using ear-EEG. *Biomedical Engineering Online* **16.** `https://biomedical-engineering-online.biomedcentral.com/articles/10.1186/s12938-017-0400-5` (Sept. 2017).

2. Huupponen, E. *et al.* Development and comparison of four sleep spindle detection methods. *Artificial intelligence in medicine* **40,** 157–170 (2007).

3. Lajnef, T. *et al.* Sleep spindle and K-complex detection using tunable Q-factor wavelet transform and morphological component analysis. *Frontiers in human neuroscience* **9,** 414 (2015).

4. Duman, F., Erdamar, A., Erogul, O., Telatar, Z. & Yetkin, S. Efficient sleep spindle detection algorithm with decision tree. *Expert Systems with Applications* **36,** 9980–9985 (2009).

5. Costa, M., Goldberger, A. L. & Peng, C.-K. Multiscale entropy analysis of complex physiologic time series. *Physical review letters* **89,** 068102 (2002).

6. Ma, Y., Shi, W., Peng, C.-K. & Yang, A. C. Nonlinear dynamical analysis of sleep electroencephalography using fractal and entropy approaches. *Sleep medicine reviews* **37,** 85–93 (2018).

7. Chung, C.-C. *et al.* Multiscale entropy analysis of electroencephalography during sleep in patients with Parkinson disease. *Clinical EEG and neuroscience* **44,** 221–226 (2013).

8. Koley, B. & Dey, D. An ensemble system for automatic sleep stage classification using single channel EEG signal. *Computers in biology and medicine* **42,** 1186–1195 (2012).

9. Zhang, X.-S., Roy, R. J. & Jensen, E. W. EEG complexity as a measure of depth of anesthesia for patients. *IEEE transactions on biomedical engineering* **48,** 1424–1433 (2001).
